# The correlation between gut microbiota and both neurotransmitters and mental disorders: A narrative review

**DOI:** 10.1097/MD.0000000000037114

**Published:** 2023-02-02

**Authors:** Amjad Mhanna, Nafiza Martini, Ghefar Hmaydoosh, George Hamwi, Mulham Jarjanazi, Ghaith Zaifah, Reem Kazzazo, Aya Haji Mohamad, Zuheir Alshehabi

**Affiliations:** a Faculty of Medicine, Tishreen University, Latakia, Syrian Arab Republic; b Stemosis for Scientific Research, Damascus, Syrian Arab Republic; c Damascus University, Faculty of Medicine, Damascus, Syrian Arab Republic; d Pediatric Surgery Resident, Pediatric Surgery Department, Aleppo University Hospital, Aleppo, Syrian Arab Republic; e Faculty of Medicine, Aleppo University, Aleppo University Hospital, Aleppo, Syrian Arab Republic; f Department of Pathology, Tishreen University Hospital, Latakia, Syrian Arab Republic.

**Keywords:** gut microbiota, neurotransmitters, mental disorders

## Abstract

The gastrointestinal tract is embedded with microorganisms of numerous genera, referred to as gut microbiota. Gut microbiota has multiple effects on many body organs, including the brain. There is a bidirectional connection between the gut and brain called the gut-brain-axis, and these connections are formed through immunological, neuronal, and neuroendocrine pathways. In addition, gut microbiota modulates the synthesis and functioning of neurotransmitters. Therefore, the disruption of the gut microbiota in the composition or function, which is known as dysbiosis, is associated with the pathogenesis of many mental disorders, such as schizophrenia, depression, and other psychiatric disorders. This review aims to summarize the modulation role of the gut microbiota in 4 prominent neurotransmitters (tryptophan and serotonergic system, dopamine, gamma-aminobutyric acid, and glutamate), as well as its association with 4 psychiatric disorders (schizophrenia, depression, anxiety disorders, and autism spectrum disorder). More future research is required to develop efficient gut-microbiota-based therapies for these illnesses.

**Key points:**

- The gut microbiomes are linked with the brain through the gut-brain axis.
- The gut microbiota has a clear impact on neurotransmitters including serotonin, dopamine, GABA, and glutamate.
- Mental disorders such as schizophrenia, depression, anxiety disorders, and autism spectrum disorder are associated with gut microbiota.
- Therapies influencing the gut microbiota including probiotics, prebiotics, and fecal microbiota transplant could help in the treatment of mental illnesses.

## 1. Introduction

### 1.1. Background

The human microbiota consists of numerous species and plays an essential role in several functions, including the digestive process, vitamin synthesis, and metabolism.^[1,2]^ A balanced microbiota population is crucial for homeostasis,^[3]^ Therefore, the abnormal composition of human microbiota may lead to several illnesses like immune system issues, infections, and neuropsychiatric disorders.^[1,4]^ The greatest number and diversity of these microorganisms are found in the gut.^[5]^ Recently, a growing body of evidence suggests that gut microbiota including bacteria, eukaryotes, and archaea has a significant role in the pathophysiology of several illnesses in many systems and organs.^[3,6]^ The brain is one of the organs that is affected by the gut microbiota through the gut-brain axis.^[3,5]^

### 1.2. Microbiota gut-brain axis

The gut-brain axis is a complex communication system connecting the gut with the brain, which works through a combination of neural, immunological and chemical signaling pathways, comprising the vagus nerve as one of the most important direct pathways. The immune system is also impacted by both the gut and the brain, with the gut microbiota playing a critical role in immune system physiology.^[7]^ Additionally, chemical substances, such as short-chain fatty acids (SCFAs) and neurotransmitters produced by the gut microbiota directly influence brain function.^[4]^

The gut-brain axis is found to be implicated in the pathophysiology of numerous mental health disorders, such as depression, anxiety and many more.^[8]^ Several studies on mice have shown that the gut microbiota and their byproducts can influence behavior in cases of autism spectrum disorder (ASD).^[9]^ In this article, we will review in more detail some of these disorders and their correlation with gut microbiota.

Understanding how the gut-brain axis works has led to the development of novel therapeutic approaches aimed at targeting the gut microbiota to improve brain disorders symptoms. Microbiome-based therapeutic interventions, including probiotics, prebiotics, fecal microbiota transplant (FMT) and dietary interventions have all been explored as potential strategies aimed to support mental well-being.^[10]^ Kang et al showed significant improvements regarding both gastrointestinal (GI) and behavioral symptoms in children with ASD, that were still detectable 2 years after discontinuing the microbiota transfer therapy treatment.^[9]^ Unravelling the intricacies of this complex dynamic system might help researchers develop novel treatments for a wide range of conditions that involve disturbances in gut-brain communication.^[4]^

### 1.3. Gut dysbiosis

Eubiosis refers to the state of equilibrium in the formulation and composition of the gut microbiome. Gut microbes are essential for the maintenance of the intestinal microenvironment, gut barrier homeostasis, motility, and immune system regulation.^[11]^ Gut dysbiosis is, therefore, defined as an alteration or disruption in the balance, composition and diversity of the gut microbiome. This imbalance can involve an abundance in the pathobionts (pro-inflammatory cytokines producing microbes), in opposition to a scarcity of the symbionts (anti-inflammatory cytokines producing microbes). Several mechanisms are integrated in the pathophysiology of gut dysbiosis, comprising an increased permeability due to decreased expression of claudin-5 and occludin (tight junction proteins linking between intestinal epithelial cells), all of which ultimately results in what is known as the leaky gut syndrome.^[12]^ Leaky gut syndrome can precipitate systemic inflammation by making it easier for the gut bacteria to enter the bloodstream.^[11]^ Another pathophysiological mechanism, in which leaky got syndrome can evoke systemic inflammation, is by disintegrating the blood-brain barrier through impairing the junction proteins in the hippocampus, striatum and frontal lobe cortex. These alterations in the brain permeability prompt a faulty displacement and increased immigration of immune cells, as well as harmful microbial metabolites toward the brain, which can consequently elevate cytokines and endocrinal stress transmitters in the brain tissue.^[12]^ As a result, gut dysbiosis is related to unpleasant health conditions, including diabetes, obesity and asthma, in addition to a wide variety of diseases affecting the GI, cardiovascular and central nervous system (CNS).^[11,12]^ The close linkage between gut dysbiosis and neuropsychiatric disorders has recently become more evident with forthcoming clinical research highlighting the coexistence of dysfunctional GI tract in patients with depression, autistic disorder, anxiety and schizophrenia.^[11,12]^ More research is still, however, required to identify the exact pathophysiology behind the route of influence in between gut dysbiosis and brain disorders.

**Figure 1.**
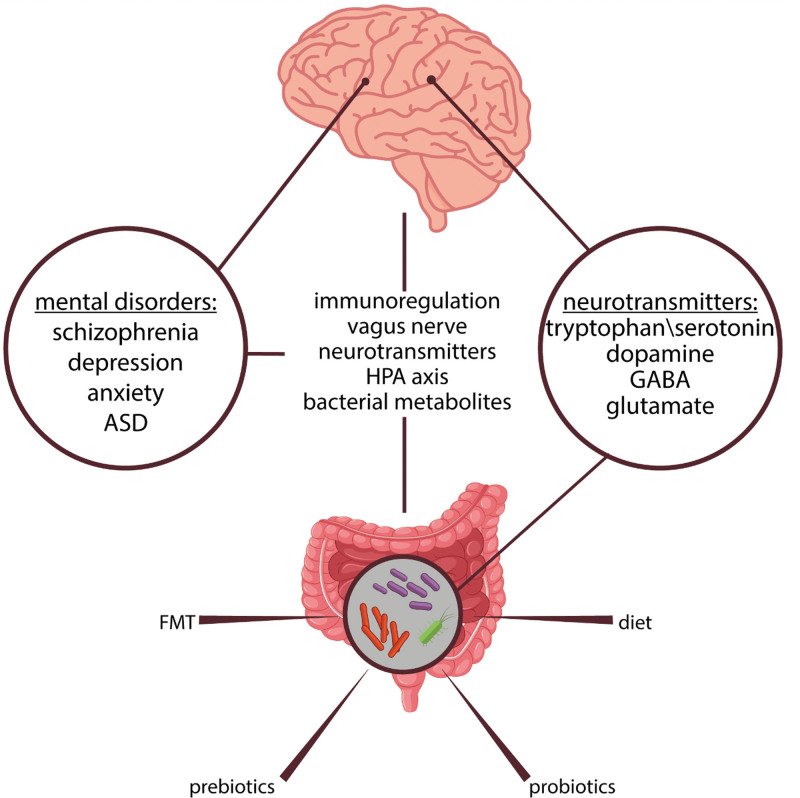
The role of gut microbiota in both neurotransmitters (including serotonin, dopamine, GABA, and glutamate), and psychiatric illnesses (including depression, anxiety, schizophrenia, and ASD) through the gut-brain axis, which comprises several pathways and mediators such as immunoregulation, the vagus nerve, the HPA axis, bacterial metabolites. Hence, therapeutic applications targeting the gut microbiota (such as FMT, diet, probiotics, and prebiotics) may give rise to more promising and efficient treatments for these disorders in the future. ASD = autism spectrum disorder, FMT = fecal microbiota transplantation, HPA axis = hypothalamic-pituitary-adrenal axis.

Based on the clear relationship between gut microbiota and the brain, we discuss in this review the role of gut microbiota in both neurotransmitters and mental disorders. We chose 4 neurotransmitters [tryptophan and serotonergic system, dopamine, gamma-aminobutyric acid (GABA), and glutamate] and 4 psychiatric illnesses (schizophrenia, depression, anxiety disorders, and ASD) (Fig. 1). These widespread disorders have a great impact on public health especially in low-income countries.

## 2. Gut microbiota and neurotransmitters

### 2.1. Gut microbiota and tryptophan\serotonergic system

Tryptophan is an essential alpha-amino acid acquired almost exclusively from a protein-rich diet.^[13,14]^ The World Health Organization advises that a total of 4 mg/kg of tryptophan is achieved daily.^[15]^ Tryptophan has the chemical formula: 2-amino-3 (1H-indol-3-yl) propanoic acid and can exist in 3 different isomeric arrangements (D, L, DL).^[13,14]^ In addition to its key role in numerous physiological processes including protein synthesis,^[13]^ maturation and regulation of immunological and neuropsychological functions,^[16]^ and maintaining homeostasis of the gut environment,^[4]^ tryptophan is also the precursor to many bioactive metabolites which physiologically influence the human body and sustain its homeostasis.^[13]^ Due to the extensive surface area of the human GI tract, a complex diverse ecosystem of approximately 100 trillion microorganisms from 1000 to 5000 various species, termed the gut microbiota, colonize the surface of the gut wall in the alimentary canal.^[7,15,16]^ The gut microbiota affects the metabolism of host tryptophan in multiple direct and indirect mechanisms; the direct methods are executed by metabolites of the resident bacteria, including SCFAs, indole derivatives, and hydrogen peroxide; indirect methods comprise immunological modulations influencing the tryptophan metabolism pathway.^[17]^ Upon digestion, dietary tryptophan is released in the small intestine, where it can be absorbed into the peripheral bloodstream to serve as a metabolic substrate to the host cells,^[14,17]^ be transformed into serotonin by the enterochromaffin cells, or degraded via the kynurenine pathway.^[14]^ The minority of unabsorbed tryptophan is processed by the gut microbiome into biomolecule derivatives called indoles, which are essential for maintaining the communication and survival of the resident bacteria.^[14,17]^ Serotonin or 5-hydroxytryptamine (5-HT) is a monoamine metabolite that is derived from tryptophan in a conversion mediated by tryptophan hydroxylase.^[17,18]^ It plays a crucial role as a neurotransmitter that regulates mood, body temperature, pain perception, food intake and appetite, circadian rhythm, sexuality, memory, and stress response.^[18,19]^ Numerous bacterial species, including *Streptococcus, Lactobacillus, Klebsiella,* and *Escherichia coli* have reportedly expressed serotonin-synthesizing properties via tryptophan synthetase mediation.^[18]^ Since 90% of serotonin synthesis occurs peripherally in the distal GI tract, it is of no surprise that forthcoming research is linking tryptophan metabolism, and serotonin host levels to the gut microbiota.^[14]^

Evidence on the effect of the gut microbiome on tryptophan metabolism is established by research on animal models depleted of gut microorganisms.^[14]^ Studies on germ-free mice revealed an increase in plasma tryptophan and brain serotonin concentration, that shifted back to normal after gut microbiota is established post-weaning, which is an expected finding since these commensal microorganisms utilize tryptophan as a metabolic substrate.^[14,17]^ Findings from studies on animal models concluded that host tryptophan availability could be limited by manipulation of the microbiome and subsequently tryptophan metabolic pathways.^[18]^ The accommodating bacteria effect on brain functions via the gut serotonin system was recognized in a study that found that spore-forming bacteria could directly stimulate serotonin biosynthesis via a complex metabolite/cell component-sustained mechanism in the enterochromaffin cells inside the mice and human GI tract.^[17,18]^

Normal host microbiome plays a protective buffer to the serotonergic system against fluctuations of its tryptophan-derived precursors, however, a study on germ-free mice exhibited that alterations in the serotonergic system could not be normalized by reinstating the microbiological ecosystem in early adolescence, suggesting that gut microbes can only execute their effects on the serotonergic system during a developmental window. Correspondingly, host serotonin fluctuations influence the gut microenvironment, because serotonin is a signaling molecule that enhances bacterial growth in specific colonies.^[17]^

It has also become clear that tryptophan and serotonin are centrally integrated in the pathogenesis of many neurological and psychiatric disorders, such as depression and anxiety.^[14]^ Hence, experiments on germ-free mice displayed relatively more anxious behavior in comparison with the control group of conventionally-raised mice, which probably suggest a humoral pathway through which, the microbiota could alter serotonergic neurotransmission in the brain.^[14,18]^ Moreover, multiple studies investigated the impact of 5-HT modulation on depression and/or anxiety using microbiome-dependent interventions.^[20]^

Additionally, Tryptophan metabolism is, to a wide extent, involved with depression; results of a study regarding tryptophan influence on affective disorders revealed improvement in mood states and a decline in depressive symptoms in individuals following a tryptophan-rich diet, in comparison with anxiety and irritability in counterparts on a low-tryptophan diet.^[19]^ Kelly et al studied the effect of gut microbes on altering kynurenine metabolism and consequently depressive features by applying FMT from depressed patients into microbiome-devoid rats; they reported depressive manners accompanied by substantial kynurenine to tryptophan ratio increase in the plasma.^[21]^ Higher susceptibility to schizophrenia and suicidal behavior is linked to specific polymorphisms in the tryptophan hydroxylase1 enzyme, plausibly due to an impairment in the tryptophan to serotonin conversion pathway. Furthermore, low levels of the serotonin metabolite: 5-hydroxyindole acetic acid in the cerebrospinal fluid were associated with suicidal and aggressive behavior.^[14]^

Understanding the precise ways gut microbiota and tryptophan metabolism crosstalk in the pathophysiology of depression might open new horizons toward more efficient treatments for this disorder, which affects over 240 million people globally.^[15]^

### 2.2. Gut microbiota and dopamine

Dopamine is a neurotransmitter generated in both the CNS and the periphery and acts by attaching to G protein-coupled receptors.^[22]^ Dopamine is delivered to the brain via the phenylalanine-tyrosine-dopa-dopamine route. The rate-limiting enzyme in this process is tyrosine hydroxylase, which hydroxylates tyrosine and produces levodopa (L-dopa) using tetrahydrobiopterin as a cofactor.^[23]^ Dopamine has a broad range of activities. It is essential for excitement, mobility, mood, and the execution of activities that involve fast decisions and learning through reward.^[24]^ There is a strong relationship between the gut microbiome and dopamine. Some gut microorganisms have been found to have neuroprotective effects on dopaminergic neurons, slowing dopamine depletion. Other bacteria, on the other hand, can have a detrimental impact by activating inflammatory responses via endotoxins, further depleting dopamine quantities.^[25]^ *Lactobacillus casei (L casei*), for example, can increase the levels of monoamines dopamine, 5-HT, and norepinephrine in the frontal brain of rats.^[26]^ *Prevotella, Bacteroides, Lactobacillus, Bifidobacterium, Clostridium, Enterococcus,* and *Ruminococcus* have roles in regulating receptors, transporters, and particular targets of the dopaminergic pathway either positively or negatively. Several investigations have shown that L-dopa supplementation allows *Enterococcus faecium* in the GI tract to convert L-dopa into dopamine. Furthermore, neuroimaging revealed that transplanting both *Enterococcus faecalis* and *E faecium* into a mouse model of Parkinson disease significantly increased the amount of striatal dopamine.^[25]^ Dopamine also affects pathogenic bacteria, such as *E coli*, which grows faster in the presence of dopamine and norepinephrine.^[27]^ In light of these facts, many interventions can be done to modify dopamine levels by affecting gut microbiota leading to several therapeutic applications on diseases caused by disruption of the healthy gut microbiota, including Parkinson disease, attention deficit hyperactivity disorder, depression, anxiety disorder and ASD.^[25]^ *L casei* intervention can ameliorate the changes in intestinal microbiota composition caused by chronic unpredictable mild stress (CUMS), indicating that *L casei* supplementation could improve dysbacteriosis caused by depression and restore the homeostasis of intestinal microbiome microecology.^[26]^ Taking *Lactobacillus plantarum* for 12 weeks decreased stress and anxiety in stressed individuals compared to a placebo group, and this was associated with alterations along the brain neurotransmitter pathways of serotonin and dopamine-norepinephrine.^[28]^ Recent genomic investigations have discovered that ephrin B6 (EPHB6) is altered in some ASD patients. EPHB6 is a potential ASD-associated gene and is a member of the ephrin family of receptor tyrosine kinases. A study using EPHB6-deficient animals revealed a new regulatory function for the gut microbiota on dopamine in the prefrontal cortex by affecting vitamin B6 levels. The study findings suggested that deficiencies in vitamin B6 reduced dopamine levels, which in turn caused social deficits and an excitation/inhibition imbalance in mice lacking the enzyme ephB6.^[29]^ According to another study, the unique alkaloid neferine, which is derived from the seed of the lotus plant, has therapeutic effects on depressed mice because it can diminish hippocampal nerve damage, reduce anti-depressant neurotransmitter secretion, and improve the structure of the gut microbiota. Particularly, *Lactobacillus* may be the gut microbial target of neferine in alleviating the symptoms of depression.^[30]^ The consumption of *Bacillus coagulans* can lessen the anxiety- and depression-like phenotypes caused by CUMS and maternal separation models in rats. The alteration of the gut-brain axis’ microbiome may be responsible for the reversal of emotional behavior after treatment with *B coagulans*.^[31]^

### 2.3. Gut microbiota and GABA

GABA is an inhibitory neurotransmitter which plays an important role in behavior, cognition, and the body response to stress, it has also been associated with several positive health effects, such as reducing anxiety and menopausal syndrome symptoms, boosting immunity, treating depression and insomnia, regulating blood pressure, fighting obesity, improving the visual cortex performance.^[32]^

The postsynaptic membrane has 3 GABA receptors (GABARs) called alpha, beta, and gamma that can recognize and bind GABA. When GABA binds to GABARs in humans, it opens ion channels at the inhibitory synapses, allowing chloride ions to enter the cell and potassium ions to exit.^[33]^ Glutamate decarboxylase catalyzes the α-decarboxylation of l-glutamate to produce GABA.^[34]^ Currently, there are numerous methods for obtaining GABA, including chemical synthesis, enriching plants, enzymatic processes, and microbial production.^[35]^ GABA can also be produced from ornithine, arginine, and putrescine, and numerous human gut bacteria have been found to contain homologous biosynthesis enzymes.^[36]^

Bacteria use neurotransmitters to communicate with the CNS and release molecules into the bloodstream that regulate physiological processes in the intestinal wall.^[37]^ The *Parabacteroides* and *Eubacterium* genera were identified as GABA producers,^[38]^ along with *Lactobacillus, Bifidobacterium, Bacteroides*,^[37]^ and *Blautia*,^[39]^ specifically *Bacteroides fragilis*.^[37]^

A recent human study showed that transplantation of the fecal microbiota from lean to obese people increased plasma GABA levels.^[38]^ And it also increased in the hippocampus and prefrontal cortex after treatment with *Lactobacillus*.^[37]^ Reduced levels of *Bifidobacterium pseudolongum* and elevated levels of *Desulfovibrio piger* and *Mucispirillum schaedleri* may be able to decrease hippocampal GABA levels.^[40]^

According to human studies to date, the gut microbiome is altered in major depression.^[41]^ The expression of GABARs was changed in the brain by *Lactobacillus rhamnosus*, which decreased depression and anxiety.^[37]^ One of the products of Blautia-dependent arginine metabolism is the gut microbial neurotransmitter GABA, its increase was linked to a lower risk of Alzheimer Disease.^[39]^ Due to its inverse relationship with GABA levels, the abundance of *M schaedleri* may be associated with the development of postpartum depression.^[40]^ Given that AD develops over a long prodromal period, it is conceivable that early interventions targeting the microbiota could be effective in treating this disorder in the future (e.g., antibiotics, psychobiotics, or gut microbiota transplantation).^[39]^

### 2.4. Gut microbiota and glutamate

Glutamate is a non-essential amino acid that is found in a wide variety of foods and natural substances including meat, fish, cheese, and vegetables.^[42,43]^ Other endogenous sources include the body production of glutamate from metabolic pathways or its release from synaptic vesicles. It is also stored in nerve cells for later use.^[44]^ Glutamate metabolic pathway is a complex biochemical process that comprises 89 metabolites, most importantly: N-acetyl-l-glutamate, δ-1-pyrroline-5-carboxylate, β-citrylglutamate, l-γ-glutamyl-l-cysteine. Glutamate is synthesized from glutamine, α-ketoglutarate, and 5-oxoproline.^[45]^ It is a crucial factor in taste perception and signal transmission in the brain. Glutamate is also the major excitatory neurotransmitter and the most concentrated amino acid in the CNS. Conversely, the major inhibitory neurotransmitter, GABA, is synthesized from glutamate.^[42,46]^ Enteric glutamate contributes significantly to the microbiota-gut-brain axis.^[47]^ Various molecules produced by gut microbiota may regulate diverse functions in the intestinal tract, such as metabolic, nutritional, and immune responses. Additionally, they can impact brain activity, leading to a microbiome-driven control of the CNS. In this scenario and because glutamate plays a role in regulating various functions along the gut-brain axis, conditions such as depression,^[48]^ Alzheimer disease, ASD,^[42]^ and other neuropsychological disorders are linked with glutamatergic signaling and the makeup of the gut microbiota.^[46]^ Multiple studies have shown that changes in gut microbiota can alter brain levels of glutamate.^[46]^ These findings suggest that the gut microbiota may affect glutamate production in the brain through enzymatic pathways and l-tryptophan metabolism is an indirect pathway through which the gut microbiota can influence glutamate pathways. Furthermore, prospective animal studies showed that microorganisms in the intestines can regulate the metabolism of d-amino acids in the brain, this finding was further supported by research on autism patients.^[42,46]^ Moreover, it has been hypothesized that plasma and fecal levels of glutamate are influenced by the compilation of the gut microbiota. Animal studies indicate that the gut microbiota can alter the ratios of hippocampal GABA/glutamate levels, which are critical for synaptic plasticity, learning, and memory mechanisms.^[42]^ Additionally, there is evidence linking changes in the gut microbiome with GI disorders such as irritable bowel syndrome and inflammatory bowel disease. In these conditions, alterations in glutamate signaling may also contribute to symptom development.^[46]^

## 3. Gut microbiota and mental disorders

### 3.1. Gut microbiota and schizophrenia

Schizophrenia is a principal cause of impairment globally, with a lifetime prevalence of 1% and a highly heterogeneous etiology.^[49–51]^ The gut microbiome has been linked to the development and maintenance of schizophrenia,^[52]^ This association has been a prominent trend in schizophrenia research for the past 50 years,^[53]^ primarily due to advances in sequencing methods.^[54]^ A recent systematic review revealed that schizophrenia often exhibits higher levels of *Prevotella* and lower levels of *Haemophilus, Bacteroides,* and *Streptococcus*.^[55]^ These gut microbes could be involved in the pathogenesis of schizophrenia by causing or exacerbating neuroinflammation due to gut dysbiosis. This could happen in multiple ways, including microbial translocation to the systemic circulation, enhanced release of cytokines, and via the vagus nerve through the cholinergic anti-inflammatory pathway.^[49,56]^ The interaction of stress and dysregulation of the gut microbiome is also important. Gut dysbiosis may increase stress sensitivity through the hypothalamic-pituitary-adrenal (HPA), a major neuroendocrine unit that regulates mood. Furthermore, stressful life events combined with the effects of the gut-brain axis may incite bidirectional complications leading to or exacerbating schizophrenia.^[49,51,57]^ The role of GABAergic transmission in schizophrenia has recently risen in significance, while gut commensal strains *Lactobacillus* and *Bifidobacterium* have been found able to produce GABA.^[58]^ Some GI hormones have been linked to cognition. The gut microbiome influences the secretion of these hormones, modifying the ecology and function of the gut microbiome.^[59]^ Several studies have linked specific clinical features of schizophrenia to unique gut microbial states, but the correlations from each study have not been consistently replicated.^[60]^ Manipulating the gut microbiome may modify the metabolism of d-amino acids, one of which is d-serine, a non-essential amino acid with antipsychotic activity and a selective full agonist of N-methyl-d-aspartate-type glutamate receptor. Decreased levels of d-serine could be associated with schizophrenia, as N-methyl-d-aspartate receptor antagonists have recently been associated with cognitive impairments.^[58,61]^ There has been significant data suggesting that the gut microbiome composition is responsible for a drug-unresponsive form of psychosis, an effect that could be alleviated with parenteral drug administration.^[62]^ Further, several studies described specific alterations in the gut microbiome composition, which co-occurred with metabolic comorbidities such as hypertension, weight gain, and diabetes. The same studies showed that an antipsychotic-induced reversal of these metabolic comorbidities correlated with changes in the gut microbiome composition.^[63]^ Finally, the association of the gut microbiome with schizophrenia could lead to alternative non-pharmaceutical approaches to treat the disorder. In particular, altering gut microbial diversity with psychobiotics can be the next promising step in schizophrenia research and clinical practice. The use of psychobiotics constitutes combining probiotics, live organisms possessing therapeutic effects, with prebiotics, food that can be processed by the probiotics in the colon, to produce metabolites that sustain the gut microbiome leading to health benefits across the gut-brain axis.^[50,56,59,63]^

### 3.2. Gut microbiota and depression

Depression is the second leading cause of disability worldwide.^[64]^ The role of the gut microbiome has been implicated in depressive disorders.^[65,66]^ Genetically speaking, changes in the gut microbiome composition could be associated with alterations in the epigenetic regulation and gene expression of receptors and mediators that are connected with depressive disorders, such as brain-derived neurotrophic factor and G protein-coupled receptors.^[65]^ A wide range of environmental, genetic, and lifestyle factors can contribute to variances in the gut microbiome composition, and even romantic couples with relatively high intimate kiss frequencies can show transient alterations and shares of salivary microbiota, emphasizing that contact with our cohabitants, including pets, may significantly shape the composition of our gut microbiome.^[67,68]^ Some studies found associations between specific alterations in the gut microbiome composition and depression. A cohort of young major depressive disorder patients was found to have an increased abundance of *Neisseria* spp. and *Prevotella nigrescens* in their salivary microbiome compared to control subjects.^[69]^ Another study found a relative abundance of *Bacteroidetes* and a reduction of *Lachnospiraceae* in depressed patients.^[70]^ Furthermore, specific bacterial genera in the gut microbiome such as *Escherichia, Enterococcus, Candida,* and *Streptococcus*, have been found to produce serotonin, a key neurotransmitter in depression.^[71]^ Meanwhile, antibiotics and poor diet can cause dysfunction of the gut microbiome leading to disparities in neurotransmitters that are involved in depressive disorders through the microbiome-gut-brain axis, possibly caused by either an increase in neuroinflammation or a decrease in neuroplasticity and neurogenesis, with these effects being mediated by the HPA axis or vagus nerve.^[66,70]^ These effects could also be mediated directly by the leakage of microbial metabolites and exogenous compounds through the disrupted gut-blood or blood-brain barriers. Innate immune responses due to digestion-resistant gliadin protein and peptides in individuals with gluten intolerance could increase the permeability of tight junctions through the increase of zonulin, which is a modulator of intercellular tight junctions and trafficking of macromolecules.^[72]^ Finally, clinical studies concerning the beneficial role of probiotics, prebiotics, and postbiotics in mental disorders have also encompassed depression. In particular, clinical studies have favored the potential of probiotics, to that of prebiotics or postbiotics, in reducing symptoms of depression.^[73]^

### 3.3. Gut microbiota and anxiety disorder

Anxiety disorders are quite common among adults. Generalized anxiety disorder (GAD) is among the most common and chronic forms of anxiety, with a current prevalence of 4% to 6% of the total population.^[74]^ GAD is associated with daily life activities impairment and is characterized by persistent and excessive worrying.^[75]^

Research has indicated that gut microbiota plays a key role in anxiety disorders through the gut-brain axis. However, in the case of anxiety, the gut microbiota affects the tryptophan-kynurenine pathway, blocking the conversion of tryptophan into serotonin, which results in serotonin depletion and the development of anxiety disorders.^[76]^

The imbalance of the gut microbiota components can act as a predisposing factor to various mental disorders, including anxiety. A longitudinal pilot study performed on GAD patients indicated the relationship between GAD and decreased gut microbiota. GAD dysbiosis is characterized by the alteration of several genera of gut microbiota. The major component of the fecal microbiota, which is *Bacteroides*, is decreased in GAD patients in comparison with healthy individuals. In addition, the 5 SCFA-producing genera (*Eubacterium rectale, Faecalibacterium, Butyricicoccus, Sutterella,* and *Lachnospira*) were decreased in GAD patients’ guts.^[77]^ In addition, animal models have shown that gut microbiota regulates stress response. Sudo et al reported that the stress hormone corticosterone and adrenocorticotropic hormone in germ-free mice were elevated, due to the activation of the HPA axis.^[78]^

Finally, the modulation of gut microbiota dysbiosis can have an important effect on anxiety. A recent study on mice indicated that the oral administration of *Lactococcus lactis* probiotic alleviated CUMS-induced anxiety and improved anxiety-like behaviors by improving gut microbiota dysbiosis through restoring the abundances of *Firmicutes* and *Bacteroidetes*. As well as reducing the serum corticosterone level, increasing serum 5-HTP levels, and restoring the central levels of serotonin.^[79]^

### 3.4. Gut microbiota and ASD

ASD is a neurodevelopmental condition noticed in children in early life.^[80]^ Many genetics and environmental factors contribute to ASD pathogenesis, but the exact pathophysiology is yet understood.^[1]^ ASD impacts social interaction and communication. The patients also have many repetitive behaviors, eye contact avoidance, and difficulties in adaptation to changing routines.^[5,81]^ According to several studies, this disorder happens in males more than females, with a ratio of 4:1.^[80]^

In addition to cognitive impairments, it is well-known that ASD children have various GI problems, such as gut dysfunction, constipation, diarrhea, and recurrent abdominal pain. The GI symptoms are closely correlated to ASD severity, and they might have a relation with dysbiosis in the gut microbiome.^[1,82]^

Recently, researchers suggest that gut microbiota is related to ASD etiology through the microbiota-gut-brain axis. Autistic children have an alteration in their bowel microbiome composition, compared to healthy children.^[1]^

For example, the GI tract of ASD patients has a considerable rise in the numbers of *Desulfovibrio* species, *Lactobacillus* species, and *Clostridium perfringens*.

Many studies show that gut microbiota alteration in ASD individuals may improve their behavior.^[2,83]^ Furthermore, the ketogenic diet (KD) may be beneficial in ASD management. The KD diet is a high-fat, appropriate-protein, low-carbohydrate diet that may be used to treat many neurological and psychiatric disorders. The mechanism behind the KD effect is still unclear, but according to animal studies, KD may improve ASD symptoms by altering the gut microbiome composition.^[83]^

In this field, many promising therapies could be helpful. For instance, probiotics and FMT demonstrate a considerable effect on GI symptom treatment in ASD, they also could improve behavior problems. However, until today, much remains to be discovered about the role of the microbiota-gut-brain axis in ASD.^[84]^

Table 1 summarize the relationship between gut microbiota and the recent 4 mental conditions.

**Table 1:** The table summarizes the evidence linking alterations in gut microbiota to several major mental disorders, including schizophrenia, depression, anxiety, and autism spectrum disorder.

	Gut microbiota and mental disorders			
	Schizophrenia	Depression	Anxiety disorder	Autism spectrum disorder (ASD)
A brief introduction	Schizophrenia is a significant cause of disability with varied causes, and gut microbiome has been linked to its development^[45–50]^	Depression is a prevalent cause of disability worldwide, with the gut microbiome being implicated in its pathogenesis^[60–62]^	Generalized anxiety disorder (GAD) is a prevalent and enduring type of anxiety, affecting 4%–6% of the total population^[70]^	Autism spectrum disorder (ASD) is a neurodevelopmental condition that affects social interaction and communication and is typically identified in early childhood^[1,5,77,78]^
Neurotransmitters involved in the mental disorders	Impairment in the tryptophan to serotonin conversion pathway is plausibly linked to schizophrenia^[9]^ Researchers suggest that d-serine could be associated with schizophrenia^[54,57]^ The role of GABAergic transmission in schizophrenia has recently risen in significance^[54]^	Tryptophan and Serotonin are centrally involved in the pathogenesis of depression^[9]^ Modifying dopamine levels may have therapeutic applications for depression^[21]^ GABA has been associated with treating depression^[31]^ Glutamatergic signaling has been linked to depression^[42,44]^	Tryptophan and serotonin also play a crucial role in the pathogenesis of anxiety^[9]^ Modifying dopamine levels can have therapeutic applications for anxiety^[21]^ GABA can also reduce anxiety^[31]^	Modifying dopamine levels can have therapeutic applications on autism^[21]^ Glutamate metabolization may be linked to autism^[38,42]^
Microbiota effect on the neurotransmitters	—Numerous bacterial species have reportedly expressed serotonin-synthesizing properties^[14]^ —Gut commensal strains *Lactobacillus* and *Bifidobacterium* have been found able to produce GABA^[54]^ —Altering gut microbial may modify the metabolism of d-amino acids^[54,57]^	—Certain bacterial genera in the gut microbiome are capable of producing serotonin^[67]^ —Changing gut microbiota can modify dopamine levels^[21]^ —*Lactobacillus rhamnosus* JB-1 has been shown to alter GABARs expression in the brain, reducing depression and anxiety^[33]^ —Changes in gut microflora may also affect brain levels of glutamate^[42]^	—Several studies have examined the impact of 5-HT modulation on depression and/or anxiety using microbiome-dependent interventions^[16]^ —Affecting gut microbiota can modify dopamine levels^[21]^ —Similar to depression, *L rhamnosus* JB-1 has been shown to alter GABARs expression in the brain, reducing anxiety^[33]^	—Gut microorganisms can either slow down or accelerate dopamine depletion^[21]^ —Multiple studies have shown that changes in gut microflora can impact brain levels of glutamate^[42]^

It outlines the key neurotransmitters involved in the pathogenesis of each disorder and highlights research showing how gut microbiota can influence these neurotransmitter pathways through effects on synthesis, metabolism, and signaling.

5-HT = 5-hydroxytryptamine, GABA = gamma-aminobutyric acid, GABARs = GABA receptors.

## 4. Recommendations for a healthier gut microbiota

To promote a healthier gut microbiota, consuming a diverse range of foods is recommended. A plant-based diet that includes a variety of fruits, vegetables, whole grains, legumes, and nuts is rich in dietary fiber, vitamins, minerals, and phytochemicals that expand the gut microbial richness.^[85]^ Fermented foods like yogurt, kefir, sauerkraut, kimchi, and miso contain probiotics that can bolster the diversity of gut microbiota and maintain a balanced gut environment.^[86]^

Reducing the intake of processed foods is also important. Highly processed foods often lack the fibers and nutrients needed for a healthy gut microbiota due to their typically poor nutritional profile; they can also lead to inflammation and create changes in the gut microbiome that can be transferred to later generations via epigenetic change.^[87]^ Consuming foods rich in prebiotic fibers nurtures a healthy gut microbiota. These foods include onions, garlic, bananas, asparagus, and whole grains.^[88]^ Sleep deprivation has been associated with alterations in gut microbiota composition; thus, maintaining good-quality sleep could positively impact the gut microbiome.^[89]^ Finally, antibiotics disturb the balance of gut microbiota and favor the selection of resistant strains of bacteria; therefore, they should be used only under the supervision of healthcare professionals.^[90]^

## 5. Conclusion

In conclusion, emerging research emphasizes the significant role of the gut microbiota in mental disorders, particularly through the gut-brain axis. Dysbiosis of the gut microbiome can impact tryptophan metabolism and serotonin availability, contributing to neuropsychiatric disorders like depression. Manipulating the gut microbiota holds promise for therapeutic interventions in mental health conditions. Additionally, the gut microbiota is involved in modulating neurotransmitters like dopamine, GABA, and glutamate, which have implications for neuropsychological disorders and GI conditions. Targeting the gut microbiome represents a promising approach for managing mental disorders such as depression, schizophrenia, anxiety disorders like GAD, and ASD. However, further research is needed to understand the underlying mechanisms and optimize microbiota-targeted interventions for these conditions.

## Acknowledgments

The authors would like to express their gratitude to Stemosis for Scientific Research, a Syria-based youth organization for scientific research for providing an enriching scientific environment that facilitated this work. In addition, the authors wish to acknowledge and thank Mhd Moamen Almouallem, from Stemosis for Scientific Research, for his invaluable assistance in reviewing and editing the manuscript. His contributions from the Stemosis team helped improve the quality of the paper.

## Author contributions

**Conceptualization:** Amjad Mhanna, Nafiza Martini, Ghefar Hmaydoosh, Ghaith Zaifah, Reem Kazzazo, Aya Haji Mohamad.

**Supervision:** Amjad Mhanna, Zuheir Alshehabi.

**Writing – original draft:** Amjad Mhanna, Nafiza Martini, Ghefar Hmaydoosh, George Hamwi, Mulham Jarjanazi, Ghaith Zaifah, Reem Kazzazo, Aya Haji Mohamad.

**Writing – review & editing:** Amjad Mhanna, Nafiza Martini, Ghefar Hmaydoosh, George Hamwi, Mulham Jarjanazi, Ghaith Zaifah, Reem Kazzazo, Aya Haji Mohamad, Zuheir Alshehabi.

## References

[1] Fattorusso A Di Genova L Battista G. Autism spectrum disorders and the gut microbiota. Nutrients. 2019;11:521. 30823414 10.3390/nu11030521 PMC6471505

[2] Hughes HK Rose D Ashwood P. The gut microbiota and dysbiosis in autism spectrum disorders. Curr Neurol Neurosci Rep. 2018;18. 10.1007/s11910-018-0887-6 PMC6855251 30251184

[3] Srikantha P Hasan Mohajeri M. The possible role of the microbiota-gut-brain-axis in autism spectrum disorder. Int J Mol Sci. 2019;20:14–9. 10.3390/ijms20092115 PMC6539237 31035684

[4] Stopińska K Radziwoń-Zaleska M Domitrz I. The microbiota-gut-brain axis as a key to neuropsychiatric disorders: a mini review. J Clin Med. 2021;10:4640. 34682763 10.3390/jcm10204640 PMC8539144

[5] Lungba RM Khan SZA Ajibawo-Aganbi U. The role of the gut microbiota and the immune system in the development of autism. Cureus. 2020;12:10–4. 10.7759/cureus.11226 PMC7707059 33269154

[6] Peretti S Mariano M Mazzocchetti C. Diet: the keystone of autism spectrum disorder? Nutr Neurosci. 2019;22:825–39. 29669486 10.1080/1028415X.2018.1464819

[7] Generoso JS Giridharan VV Lee J. The role of the microbiota-gut-brain axis in neuropsychiatric disorders. Braz J Psychiatry. 2021;43:293–305. 32667590 10.1590/1516-4446-2020-0987 PMC8136391

[8] Gomaa EZ. Human gut microbiota/microbiome in health and diseases: a review. Antonie Van Leeuwenhoek. 2020;113:2019–40. 33136284 10.1007/s10482-020-01474-7

[9] Kang DW Adams JB Coleman DM. Long-term benefit of microbiota transfer therapy on autism symptoms and gut microbiota. Sci Rep. 2019;9:1–9. 30967657 10.1038/s41598-019-42183-0 PMC6456593

[10] Kang DW Adams JB Gregory AC. Microbiota transfer therapy alters gut ecosystem and improves gastrointestinal and autism symptoms: an open-label study. Microbiome. 2017;5:1–16. 28122648 10.1186/s40168-016-0225-7 PMC5264285

[11] Eiche TP Mohajeri MH. Overlapping mechanisms of action of brain-active bacteria and bacterial metabolites in the pathogenesis of common brain diseases. Nutrients. 2022;14. 10.3390/nu14132661 PMC9267981 35807841

[12] Anand N Gorantla VR Chidambaram SB. The role of gut dysbiosis in the pathophysiology of neuropsychiatric disorders. Cells. 2023;12:54–30. 10.3390/cells12010054 PMC9818777 36611848

[13] Li D Yu S Long Y. Tryptophan metabolism: mechanism-oriented therapy for neurological and psychiatric disorders. Front Immunol. 2022;13:1–18. 10.3389/fimmu.2022.985378 PMC9496178 36159806

[14] Roth W Zadeh K Vekariya R. Tryptophan metabolism and gut-brain homeostasis. Int J Mol Sci. 2021;22:2973–23. 33804088 10.3390/ijms22062973 PMC8000752

[15] Liaqat H Parveen A Kim SY. Neuroprotective natural products’ regulatory effects on depression via gut–brain axis targeting tryptophan. Nutrients. 2022;14:3270. 36014776 10.3390/nu14163270 PMC9413544

[16] Rathour D Shah S Khan S. Role of gut microbiota in depression: understanding molecular pathways, recent research, and future direction. Behav Brain Res. 2023;436:114081. 36037843 10.1016/j.bbr.2022.114081

[17] Lukić I Ivković S Mitić M. Tryptophan metabolites in depression: modulation by gut microbiota. Front Behav Neurosci. 2022;16:1–17. 10.3389/fnbeh.2022.987697 PMC9510596 36172468

[18] Gao K Mu CL Farzi A. Tryptophan metabolism: a link between the gut microbiota and brain. Adv Nutr. 2020;11:709–23. 31825083 10.1093/advances/nmz127 PMC7231603

[19] Correia AS Vale N. Tryptophan metabolism in depression: a narrative review with a focus on serotonin and kynurenine pathways. Int J Mol Sci. 2022;23:8493. 35955633 10.3390/ijms23158493 PMC9369076

[20] Huang F Wu X. Brain neurotransmitter modulation by gut microbiota in anxiety and depression. Front Cell Dev Biol. 2021;9:1–6. 10.3389/fcell.2021.649103 PMC7991717 33777957

[21] Kelly JR Borre Y O’ Brien C. Transferring the blues: depression-associated gut microbiota induces neurobehavioural changes in the rat. J Psychiatr Res. 2016;82:109–18. 27491067 10.1016/j.jpsychires.2016.07.019

[22] Klein MO Battagello DS Cardoso AR. Dopamine: functions, signaling, and association with neurological diseases. Cell Mol Neurobiol. 2019;39:31–59. 30446950 10.1007/s10571-018-0632-3 PMC11469830

[23] Wang Y Tong Q Ma S-R. Oral berberine improves brain dopa/dopamine levels to ameliorate Parkinson’s disease by regulating gut microbiota. Signal Transduct Target Ther. 2021;6. 10.1038/s41392-020-00456-5 PMC7902645 33623004

[24] Speranza L Di Porzio U Viggiano D. Dopamine: the neuromodulator of long-term synaptic plasticity, reward and movement control. Cells. 2021;10:735. 33810328 10.3390/cells10040735 PMC8066851

[25] Hamamah S Aghazarian A Nazaryan A. Role of microbiota-gut-brain axis in regulating dopaminergic signaling. Biomedicines. 2022;10:436. 35203645 10.3390/biomedicines10020436 PMC8962300

[26] Gu F Wu Y Liu Y. Lactobacillus casei improves depression-like behavior in chronic unpredictable mild stress-induced rats by the BDNF-TrkB signal pathway and the intestinal microbiota. Food Funct. 2020;11:6148–57. 32578646 10.1039/d0fo00373e

[27] Strandwitz P. Neurotransmitter modulation by the gut microbiota. Brain Res. 2018;1693:128–33. 29903615 10.1016/j.brainres.2018.03.015 PMC6005194

[28] Liu G Chong HX Chung FYL. *Lactobacillus plantarum* DR7 modulated bowel movement and gut microbiota associated with dopamine and serotonin pathways in stressed adults. Int J Mol Sci. 2020;21:4608–16. 32610495 10.3390/ijms21134608 PMC7370301

[29] Li Y Luo Z-Y Hu Y-Y. The gut microbiota regulates autism-like behavior by mediating vitamin B6homeostasis in EphB6-deficient mice. Microbiome. 2020;8:1–23. 32819434 10.1186/s40168-020-00884-z PMC7441571

[30] Dong Z Xie Q Xu F. Neferine alleviates chronic stress-induced depression by regulating monoamine neurotransmitter secretion and gut microbiota structure. Front Pharmacol. 2022;13:1–11. 10.3389/fphar.2022.974949 PMC9479079 36120376

[31] Satti S Palepu MSK Singh AA. Anxiolytic- and antidepressant-like effects of *Bacillus coagulans* Unique IS-2 mediate via reshaping of microbiome gut-brain axis in rats. Neurochem Int. 2023;163:105483. 36641109 10.1016/j.neuint.2023.105483

[32] Cui Y Miao K Niyaphorn S. Production of gamma-aminobutyric acid from lactic acid bacteria: a systematic review. Int J Mol Sci. 2020;21. 10.3390/ijms21030995 PMC7037312 32028587

[33] Sarasa SB Mahendran R Muthusamy G. A brief review on the non-protein amino acid, gamma-amino butyric acid (GABA): its production and role in microbes. Curr Microbiol. 2020;77:534–44. 31844936 10.1007/s00284-019-01839-w

[34] Xu N Wei L Liu J. Biotechnological advances and perspectives of gamma-aminobutyric acid production. World J Microbiol Biotechnol. 2017;33:0. 10.1007/s11274-017-2234-5 28247260

[35] Luo H Liu Z Xie F. Microbial production of gamma-aminobutyric acid: applications, state-of-the-art achievements, and future perspectives. Crit Rev Biotechnol. 2021;41:491–512. 33541153 10.1080/07388551.2020.1869688

[36] Quillin SJ Tran P Prindle A. Potential roles for gamma-aminobutyric acid signaling in bacterial communities. Bioelectricity. 2021;3:120–5. 34476387 10.1089/bioe.2021.0012 PMC8380936

[37] Dicks LMT. Gut bacteria and neurotransmitters. Microorganisms. 2022;10:1969. 36144440 10.3390/microorganisms10091838 PMC9504309

[38] Strandwitz P Kim KH Terekhova D. GABA-modulating bacteria of the human gut microbiota. Nat Microbiol. 2019;4:396–403. 30531975 10.1038/s41564-018-0307-3 PMC6384127

[39] Zhuang Z Yang R Wang W. Associations between gut microbiota and Alzheimer’s disease, major depressive disorder, and schizophrenia. J Neuroinflammation. 2020;17:1–9. 33008395 10.1186/s12974-020-01961-8 PMC7532639

[40] Tian XY Xing JW Zheng QQ. 919 syrup alleviates postpartum depression by modulating the structure and metabolism of gut microbes and affecting the function of the hippocampal GABA/Glutamate system. Front Cell Infect Microbiol. 2021;11:694443. 34490139 10.3389/fcimb.2021.694443 PMC8417790

[41] Dinan TG Cryan JF. The microbiome-gut-brain axis in health and disease. Gastroenterol Clin North Am. 2017;46:77–89. 28164854 10.1016/j.gtc.2016.09.007

[42] Chang CH Lin CH Lane HY. D-glutamate and gut microbiota in Alzheimer’s disease. Int J Mol Sci. 2020;21:1–17. 10.3390/ijms21082676 PMC7215955 32290475

[43] Briguglio M Dell’Osso B Panzica G. Dietary neurotransmitters: a narrative review on current knowledge. Nutrients. 2018;10:591–15. 29748506 10.3390/nu10050591 PMC5986471

[44] Hackett JT Ueda T. Glutamate release. Neurochem Res. 2015;40:2443–60. 26012367 10.1007/s11064-015-1622-1

[45] Yelamanchi SD Jayaram S Thomas JK. A pathway map of glutamate metabolism. J Cell Commun Signal. 2016;10:69–75. 26635200 10.1007/s12079-015-0315-5 PMC4850134

[46] Baj A Moro E Bistoletti M. Glutamatergic signaling along the microbiota-gut-brain axis. Int J Mol Sci. 2019;20:1482. 30934533 10.3390/ijms20061482 PMC6471396

[47] Montanari M Martella G Bonsi P. Autism spectrum disorder: focus on glutamatergic neurotransmission. Int J Mol Sci. 2022;23:3861. 35409220 10.3390/ijms23073861 PMC8998955

[48] Onaolapo AY Onaolapo OJ. Glutamate and depression: reflecting a deepening knowledge of the gut and brain effects of a ubiquitous molecule. World J Psychiatry. 2021;11:297–315. 34327123 10.5498/wjp.v11.i7.297 PMC8311508

[49] Rantala MJ Luoto S Borráz-León JI. Schizophrenia: the new etiological synthesis. Neurosci Biobehav Rev. 2022;142:104894. 36181926 10.1016/j.neubiorev.2022.104894

[50] Munawar N Ahmad A Anwar MA. Modulation of gut microbial diversity through non-pharmaceutical approaches to treat schizophrenia. Int J Mol Sci. 2022;23:2625. 35269766 10.3390/ijms23052625 PMC8910761

[51] Vafadari B. Stress and the role of the gut – brain axis in the pathogenesis of schizophrenia: a literature review. Int J Mol Sci. 2021;22:9747. 34575911 10.3390/ijms22189747 PMC8471971

[52] Tsamakis K Galinaki S Alevyzakis E. Gut microbiome: a brief review on its role in schizophrenia and first episode of psychosis. Microorganisms. 2022;10:1121–14. 35744639 10.3390/microorganisms10061121 PMC9227193

[53] Sabe M Pillinger T Kaiser S. Neuroscience and biobehavioral reviews half a century of research on antipsychotics and schizophrenia: a scientometric study of hotspots, nodes, bursts, and trends. Neurosci Biobehav Rev. 2022;136:104608. 35303594 10.1016/j.neubiorev.2022.104608

[54] Ghorbani M Rajandas H Parimannan S. Understanding the role of gut microbiota in the pathogenesis of schizophrenia. Psychiatr Genet. 2021;31:39–49. 33252574 10.1097/YPG.0000000000000270

[55] McGuinness AJ Davis JA Dawson SL. OPEN A systematic review of gut microbiota composition in observational studies of major depressive disorder, bipolar disorder and schizophrenia. Molecular psychiatry. 2022;27:1920–35. 35194166 10.1038/s41380-022-01456-3 PMC9126816

[56] Klein-petersen AW Köhler-forsberg O Benros ME. Infections, antibiotic treatment and the microbiome in relation to schizophrenia. Schizophr Res. 2019;234:71–7. 31859119 10.1016/j.schres.2019.11.033

[57] Mikulska J Juszczyk G Gawrońska-Grzywacz M. Brain sciences HPA Axis in the pathomechanism of depression and schizophrenia: new therapeutic strategies based on its participation. Brain Sciences. 2021;11:1298. 34679364 10.3390/brainsci11101298 PMC8533829

[58] Patrono E Svoboda J Stuchlík A. Schizophrenia, the gut microbiota, and new opportunities from optogenetic manipulations of the gut – brain axis. Behav Brain Funct. 2021;17:7. 34158061 10.1186/s12993-021-00180-2 PMC8218443

[59] Bioque M González-Rodríguez A Garcia-Rizo C. Targeting the microbiome-gut-brain axis for improving cognition in schizophrenia and major mood disorders: a narrative review. Prog Neuropsychopharmacol Biol Psychiatry. 2021;105:110130. 33045322 10.1016/j.pnpbp.2020.110130

[60] Nocera A Nasrallah HA. The association of the gut microbiota with clinical features in schizophrenia. Behav Sci (Basel). 2022;12:89. 35447661 10.3390/bs12040089 PMC9025473

[61] Taniguchi K Sawamura H Ikeda Y. D-Amino acids as a biomarker in schizophrenia. Diseases. 2022;10:9. 35225861 10.3390/diseases10010009 PMC8883943

[62] Seeman MV. The gut microbiome and treatment-resistance in schizophrenia. Psychiatr Q. 2020;91:127–36. 31781943 10.1007/s11126-019-09695-4

[63] Singh R Stogios N Smith E. Gut microbiome in schizophrenia and antipsychotic-induced metabolic alterations: a scoping review. Ther Adv Psychopharmacol. 2022;12:204512532210965. 10.1177/20451253221096525 PMC9118432 35600753

[64] Capuco A Urits I Hasoon J. Gut microbiome dysbiosis and depression: a comprehensive review. Curr Pain Headache Rep. 2020;24:1–14. 32506238 10.1007/s11916-020-00871-x

[65] Zalar B Haslberger A Peterlin B. The role of microbiota in depression – a brief review. Psychiatr Danub. 2018;30:136–41. 10.24869/psyd.2018.136 29930222

[66] Liang S Wu X Hu X. Recognizing depression from the microbiota–gut–brain axis. Int J Mol Sci. 2018;19. 10.3390/ijms19061592 PMC6032096 29843470

[67] Kort R Caspers M van de Graaf A. Shaping the oral microbiota through intimate kissing. Microbiome. 2014;2:1–8. 25408893 10.1186/2049-2618-2-41 PMC4233210

[68] Song SJ Lauber C Costello EK. Cohabiting family members share microbiota with one another and with their dogs. Elife. 2013;2:1–22. 10.7554/eLife.00458 PMC3628085 23599893

[69] Scassellati C Marizzoni M Cattane N. The complex molecular picture of gut and oral microbiota–brain-depression system: what we know and what we need to know. Front Psychiatry. 2021;12:1–16. 10.3389/fpsyt.2021.722335 PMC8607517 34819883

[70] Luca M Di Mauro M Di Mauro M. Gut microbiota in Alzheimer’s disease, depression, and type 2 diabetes mellitus: the role of oxidative stress. Oxid Med Cell Longev. 2019;2019. 10.1155/2019/4730539 PMC6501164 31178961

[71] Halverson T Alagiakrishnan K. Gut microbes in neurocognitive and mental health disorders. Ann Med. 2020;52:423–43. 32772900 10.1080/07853890.2020.1808239 PMC7877977

[72] Obrenovich MEM. Leaky gut, leaky brain? Microorganisms. 2018;6:1–13. 10.3390/microorganisms6040107 PMC6313445 30340384

[73] Chudzik A Orzyłowska A Rola R. Probiotics, prebiotics and postbiotics on mitigation of depression symptoms: modulation of the brain–gut–microbiome axis. Biomolecules. 2021;11. 10.3390/biom11071000 PMC8301955 34356624

[74] Mitrea L Nemeş SA Szabo K. Guts imbalance imbalances the brain: a review of gut microbiota association with neurological and psychiatric disorders. Front Med. 2022;9:1–21. 10.3389/fmed.2022.813204 PMC9009523 35433746

[75] Chen YH Bai J Wu D. Association between fecal microbiota and generalized anxiety disorder: severity and early treatment response. J Affect Disord. 2019;259:56–66. 31437702 10.1016/j.jad.2019.08.014

[76] Thangaleela S Sivamaruthi BS Kesika P. Role of probiotics and diet in the management of neurological diseases and mood states: a review. Microorganisms. 2022;10:2268. 36422338 10.3390/microorganisms10112268 PMC9696277

[77] Jiang HY Zhang X Yu ZH. Altered gut microbiota profile in patients with generalized anxiety disorder. J Psychiatr Res. 2018;104:130–6. 30029052 10.1016/j.jpsychires.2018.07.007

[78] Sudo N Chida Y Aiba Y. Postnatal microbial colonization programs the hypothalamic-pituitary-adrenal system for stress response in mice. J Physiol. 2004;558:263–75. 15133062 10.1113/jphysiol.2004.063388 PMC1664925

[79] Gao K Farzi A Ke X. Oral administration of: *Lactococcus lactis* WHH2078 alleviates depressive and anxiety symptoms in mice with induced chronic stress. Food Funct. 2022;13:957–69. 35006225 10.1039/d1fo03723d

[80] Lord C Brugha TS Charman T. Autism spectrum disorder. Nat Rev Dis Prim. 2020;6:5. 31949163 10.1038/s41572-019-0138-4 PMC8900942

[81] Gyawali S Patra BN. Autism spectrum disorder: trends in research exploring etiopathogenesis. Psychiatry Clin Neurosci. 2019;73:466–75. 31077508 10.1111/pcn.12860

[82] Ristori MV Quagliariello A Reddel S. Autism, gastrointestinal symptoms and modulation of gut microbiota by nutritional interventions. Nutrients. 2019;11:2812–21. 31752095 10.3390/nu11112812 PMC6893818

[83] Li Q Liang J Fu N. A ketogenic diet and the treatment of autism spectrum disorder. Front Pediatr. 2021;9:1–7. 10.3389/fped.2021.650624 PMC8146910 34046374

[84] Saurman V Margolis KG Luna RA. Autism spectrum disorder as a brain-gut-microbiome axis disorder. Dig Dis Sci. 2020;65:818–28. 32056091 10.1007/s10620-020-06133-5 PMC7580230

[85] Heiman ML Greenway FL. A healthy gastrointestinal microbiome is dependent on dietary diversity. Mol Metab. 2016;5:317–20. 27110483 10.1016/j.molmet.2016.02.005 PMC4837298

[86] Leeuwendaal NK Stanton C O’toole PW. Fermented foods, health and the gut microbiome. Nutrients. 2022;14:1527–26. 35406140 10.3390/nu14071527 PMC9003261

[87] Shi Z. Gut microbiota: an important link between western. Nutrients. 2019;11:2287–12. 31554269 10.3390/nu11102287 PMC6835660

[88] Fu J Zheng Y Gao Y. Dietary fiber intake and gut microbiota in human health. Microorganisms. 2022;10:2507–18. 36557760 10.3390/microorganisms10122507 PMC9787832

[89] Karl JP Whitney CC Wilson MA. Severe, short-term sleep restriction reduces gut microbiota community richness but does not alter intestinal permeability in healthy young men. Sci Rep. 2023;13:1–9. 36604516 10.1038/s41598-023-27463-0 PMC9816096

[90] Patangia DV Anthony Ryan C Dempsey E. Impact of antibiotics on the human microbiome and consequences for host health. Microbiologyopen. 2022;11:1–23. 10.1002/mbo3.1260 PMC8756738 35212478

